# Botulinum Neurotoxin Detection and Differentiation by Mass Spectrometry

**DOI:** 10.3201/eid1110.041279

**Published:** 2005-10

**Authors:** John R. Barr, Hercules Moura, Anne E. Boyer, Adrian R. Woolfitt, Suzanne R. Kalb, Antonis Pavlopoulos, Lisa G. McWilliams, Jurgen G. Schmidt, Rodolfo A. Martinez, David L. Ashley

**Affiliations:** * Centers for Disease Control and Prevention, Atlanta, Georgia, USA; † Battelle Memorial Institute, Atlanta, Georgia, USA; ‡ Los Alamos National Laboratory, Los Alamos, New Mexico, USA

**Keywords:** bioterrorism, botulism, mass spectrometry, botulinum neurotoxin, research

## Abstract

Botulinum neurotoxins (BoNTs) are proteases that cleave specific cellular proteins essential for neurotransmitter release. Seven BoNT serotypes (A–G) exist; 4 usually cause human botulism (A, B, E, and F). We developed a rapid, mass spectrometry–based method (Endopep-MS) to detect and differentiate active BoNTs A, B, E, and F. This method uses the highly specific protease activity of the toxins with target peptides specific for each toxin serotype. The product peptides derived from the endopeptidase activities of BoNTs are detected by matrix-assisted laser-desorption ionization time-of-flight mass spectrometry. In buffer, this method can detect toxin equivalents of as little as 0.01 mouse lethal dose (MLD)_50_ and concentrations as low as 0.62 MLD_50_/mL. A high-performance liquid chromatography–tandem mass spectrometry method for quantifying active toxin, where the amount of toxin can be correlated to the amount of product peptides, is also described.

## Abstract 2

A new rapid, mass spectrometry-based method to detect and differentiate botulinal neurotoxins is described.

Botulinum neurotoxins (BoNTs) are the most toxic substances known (*1*). They are produced under anaerobic conditions by strains of *Clostridium botulinum*, *C. butyricum*, and *C. baratii* (*1*). Intoxication with 1 of the 7 distinct serotypes of BoNT (A–G) causes botulism. One of 4 serotypes of BoNT (A, B, E, and F) is usually the cause of botulism in humans. BoNTs are zinc metalloproteases that cleave and inactivate specific cellular proteins essential for the release of the neurotransmitter acetylcholine (Figure 1). BoNT-A, -C, and -E cleave SNAP (synaptosomal-associated protein)-25; BoNT-B, -D, -F, and -G cleave synaptobrevin 2 (also called VAMP 2). Of the serotypes, only 1, BoNT-C, cleaves >1 site on a specific protein. In addition to cleaving SNAP-25, BoNT-C also cleaves syntaxin (*1*).

**Figure 1:**
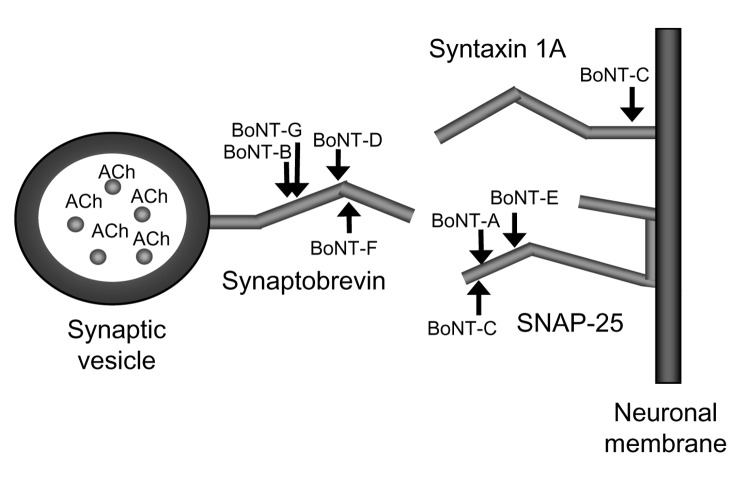
Synaptobrevin on the synaptic vesicle must interact with syntaxin and SNAP (synaptosomal-associated protein)-25 on the neuronal membrane for fusion to occur, which allows the nerve impulse to be delivered across the synaptic junction. The botulinum neurotoxin serotypes cleave the peptide bonds at specific sites on the 3 proteins, as indicated. Cleavage of any 1 of these proteins prevents vesicle membrane docking and nerve impulse transmission.

Current methods for detecting BoNT include a mouse bioassay (*2**–**4*) and an enzyme-linked immunosorbent assay (ELISA) (*5**,**6*). The mouse bioassay is the accepted standard and is the only widely accepted method for detecting BoNT (*2**–**5*). In this assay, mice receiving an intraperitoneal injection containing a sample with more than a minimum lethal dose show symptoms of botulinum intoxication and die (*2**,**4*). Many institutional animal care and use committees, including that of the Centers for Disease Control and Prevention, require mice to be euthanized after the onset of severe symptoms. In the mouse bioassay, when the injected dose is high, mice typically develop signs of botulism within 8 hours. At lower doses, mice are affected more slowly; hence, mice are observed for 4 days before a negative result is recorded. The mouse bioassay can also be used to differentiate BoNT serotypes (*4*). Mixtures of neutralizing antibodies are given to mice in conjunction with the sample. Mice receiving the appropriate anti-BoNT serotype antibody are asymptomatic and survive, while mice treated with the other serotype antibodies show symptoms of botulism (*4*). The mouse bioassay measures active toxin and is sensitive. The absolute amount of toxin detected in the mouse bioassay is not well defined but is thought to be 10–20 pg/mL for BoNT A (*7**,**8*). The main disadvantage of the mouse bioassay is that it requires euthanizing many animals. It also requires several days to determine the toxin level and type (*4**–**6*). Special animal facilities are also required, personal hazards are associated with injecting animals (*5**,**6*), and some clostridia produce nonbotulinal toxins that also kill mice (*5*).

The ELISA is more rapid than the mouse bioassay, but it is not a functional assay. It recognizes protein antigenic sites and in general is somewhat less sensitive than the mouse bioassay (*5**,**6*). The ELISA was validated for detecting BoNT produced in cooked meat medium and tryptone peptone glucose yeast extract (TPGY). The test was designed to detect and differentiate BoNT serotypes A, B, E, and F in a 1-day test and has the sensitivity of ≈10 mouse lethal dose (MLD)_50_/mL (*5**,**6*). In a recent study, the ELISA performed well in most laboratories at the 100 MLD_50_/mL and 10,000 MLD_50_/mL levels (*5*). In this study, some cross-reactivity occurred among BoNT cultures and with nonbotulinum cultures (*5*). At 100 MLD_50_/mL, a >7% false-negative rate in TPGY was observed; at 10,000 MLD_50_/mL, a 1.5% false-positive rate for BoNT-A and a 28.6% false-positive rate for BoNT-F occurred (*5*). The ELISA is currently used primarily as a fast screening technique, and results are verified by the mouse bioassay (*5*).

Several in vitro assays have been developed to detect the activities of the different BoNT serotypes (*7**–**14*). This approach has led to methods that are based on the natural substrates that are cleaved by the BoNTs and use fluorescence to detect toxin activity. These types of assays are >2 orders of magnitude less sensitive than the mouse bioassay and have not been proven for use with environmental, food, or clinical samples. They are also prone to giving false-positive results in samples that contain proteases because the specific site of cleavage cannot easily be determined in a fluorescence-based assay (*15*). Another method that combines immunoaffinity chromatography with specific antibodies for cleavage products has been successful in detecting BoNT B in some foods at lower detection limits than the mouse bioassay (*10*).

We introduce here the concept and preliminary data on a new, rapid, mass spectrometry–based, functional method for detecting, differentiating, and quantifying 4 BoNT serotypes. This method is based on both the unusual endopeptidase activities of these enzymes and specific detection of the unique peptide products by mass spectrometry (Endopep-MS). Because each BoNT serotype has a unique cleavage site on a unique peptide, the mass-specific product peptides detected by mass spectrometry differentiate active BoNT serotypes. Substrate peptides that are specific for each serotype are incubated with BoNT; then serotype-specific product peptides are detected by either matrix-assisted laser-desorption ionization time-of-flight mass spectrometry (MALDI-TOF-MS) or by high-performance liquid chromatography (HPLC)-electrospray ionization-tandem mass spectrometry (HPLC-ESI/MS/MS). Thus, this method combines the biologic specificity of the BoNT enzymatic activity with the unparalleled detection specificity of mass spectrometry.

## Methods

### Materials

**Figure 2:**
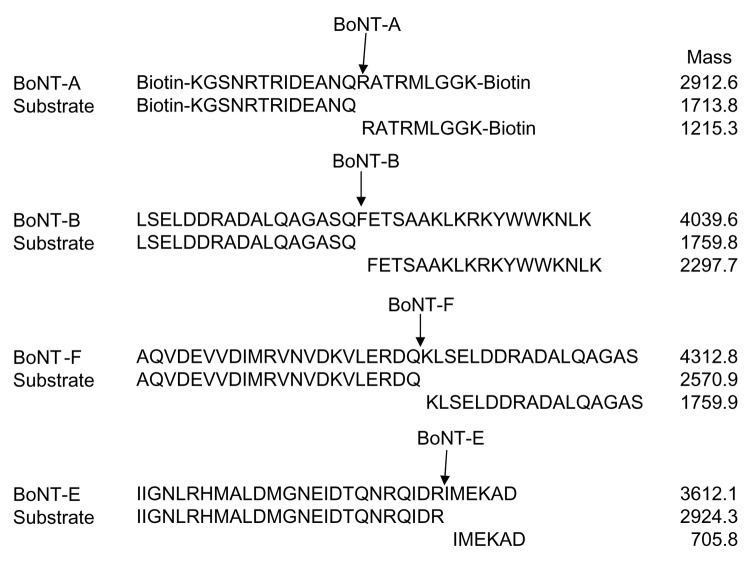
Substrate peptide sequences, the botulinum neurotoxin (BoNT) serotype predicted cleavage product sequences, and masses of the substrate and product peptides. Peptides for BoNT-A and -E were derived from the human SNAP (synaptosomal-associated protein)-25 protein. The substrate peptide for BoNT A, 187-SNKTRIDEANQRATKML-203, was modified to biotin(ε)-KG(K189->R and K201->R)GGK-(ε)Biotin. The BoNT-E substrate sequence was also from human SNAP-25 (156–186). Substrate peptides for BoNT-B and -F are from human synaptobrevin 2; the BoNT-B substrate (3) is from 59–93 in the sequence and the BoNT-F substrate is from 35–74.

BoNT complex toxins were purchased from Metabiologics (Madison, WI, USA) and were provided at 1 mg/mL total protein in 50 mmol/L sodium citrate buffer, pH 5.5. The toxin activities in MLD_50_/mg protein were 3.6 × 10^7^ BoNT-A, 1.6 × 10^7^ BoNT-B, 2.8 × 10^7^ BoNT-E, and 5.5 × 10^7^ BoNT-F. All reagents were from Sigma-Aldrich (St. Louis, MO, USA), except where indicated. HPLC-purified peptide substrates were synthesized by Los Alamos National Laboratory (Los Alamos, NM, USA). Figure 2 shows the peptide sequences used to detect and differentiate each BoNT serotype along with the specific cleavage products and their masses.

### Peptide Cleavage Reactions

For the Endopep-MS method, BoNT serotypes A, B, E, and F endopeptidase activities were determined in 20-μL volumes of buffer containing 0.05 mol/L Hepes (pH 7.3), 25 mmol/L dithiothreitol (DTT), 20 mmol/L ZnCl_2_, 1 mg/mL bovine serum albumin (BSA), and the target peptides, at 1 nmol each. Specific BoNT serotype complexes were added at various concentrations and incubated at 37°C from 2 h to 16 h. Control tubes without BoNT were run at the same time as BoNT cleavage reactions and served as an analytic blank. The analytic sensitivity of the reaction was tested by diluting the toxin in Hepes reaction buffer to 100, 10, 1, 0.1, and 0.01 MLD_50_/μL. An aliquot (1 μL) of each dilution was added to 19 μL reaction buffer containing specific peptides 1–4.

### Multiplexing Reactions

Endopeptidase reactions were multiplexed by adding all 4 peptides (*1**–**4*) at 1 nmol each to the reaction buffer described above and incubating for 2 h at 37°C. Each BoNT serotype was added to separate reaction mixtures. Control tubes with no BoNT were always run at the same time to serve as an analytic blank. High levels of BoNT (200 ng/20 μL reaction) were used for each serotype to look for cross-reactivity between any of the BoNT serotypes. No cross-reactivity between the various BoNT serotypes was observed.

### Detection Limits in MLD_50_/mL

Larger volume reactions were run to test the sensitivity in mouse LD_50_/mL. BoNT serotype A, B, E, or F complexes ranging from 100 to 0.31 MLD_50_ were spiked in 1 mL of deionized water. A 168-μL aliquot was spiked with a 10× reaction buffer and peptide solution to yield final concentrations identical to those above. The target peptides used for this experiment were the same as above, except minor modification to the BoNT A and B substrate peptides were used since they showed slightly more activity than the previous peptides. These peptides were Biotin-(ε)- KGSNRTRIDEGNQRATR(Nle)LGGK-(ε)-Biotin for BoNT A and LSELDDRADALQAGASQFESSAAKLKRKYWWKNLK for BoNT B. The final reaction volumes were 200 μL. One set of reactions was allowed to proceed for 4 h, and a second set proceeded for 16 h. The longer reaction times were used to enhance the amount of product peptide produced and, therefore, lower the detection limits. For MALDI-TOF analysis, 2 μL of the 200-μL reaction mixture was mixed with the matrix and analyzed as discussed in the MALDI-TOF-MS section. For HPLC-ESI/MS/MS analysis, 50 μL of the 200-μL reaction mixture was injected on the instrument.

### MALDI-TOF-MS Analysis

Specific cleavage products were detected by mass spectrometry. For all experiments, the reaction mixture, at the incubation times indicated, was added to alpha-cyano-4-hydroxy cinnamic acid (CHCA) at 5 mg/mL in 50% acetonitrile, 0.1% trifluoroacetic acid, and 1 mmol/L ammonium citrate (CHCA matrix), at a ratio of 1:5 or 1:10. This mixture was applied at 0.5 μL per spot to a 192-spot stainless steel MALDI plate (Applied Biosystems, Framingham, MA, USA). Mass spectra of each spot were obtained by scanning 650–4,500 *m/z* in MS-positive ion reflectron mode on a Model 4700 MALDI-TOF-TOF-MS Proteomics Analyzer (Applied Biosystems). The instrument used a nitrogen laser at 337 nm, and each spectrum was an average of 2,400 laser shots.

### HPLC-ESI/MS/MS Analysis

The HPLC-ESI/MS/MS system consisted of an API4000 triple quadrupole mass spectrometer with a TurboIonSpray interface (Applied Biosystems, Toronto, Canada) and a Shimadzu (Kyoto, Japan) liquid chromatograph. We used Luna C18 (Phenomenex, Torrance, CA, USA) columns (150 mm × 1 mm internal diameter, 5-μm particles). Solvents were A: H_2_O with 1% (vol/vol) formic acid and B: 80:20 acetonitrile:H_2_O plus 1% (vol/vol) formic acid. Peptides were eluted with a linear gradient of 0% to 80% solvent B in 25 min, at 50 μL/min. A parallel column format was used, giving a cycle time of 34 min. Tandem MS was performed by monitoring precursor to product transitions under individually optimized conditions, typically from the most abundant [M+nH]^n+^ precursor ion to an ammonium ion. For BoNT-A, the N-terminal product 1699.9 *m/z*, triply charged ion (567.5 *m/z*) fragmenting to 84 *m/z*, was monitored. For BoNT-B, the doubly charged ion (880.7 *m/z*) fragmenting to 84 *m/z* was monitored. For BoNT-E, the C-terminal product (705.8 *m/z*), doubly charged ion 353.7 → 84.0 amu, was monitored. For BoNT-F, the triply charged ion (587.6 *m/z*) fragmenting to 84.1 *m/z* was monitored. All reaction runs and transitions were monitored for the entire 32-min run time. Isotopically labeled product peptides for BoNT-A (432 *m/z* → 70 *m/z* and 368.8 *m/z* → 70 *m/z*) were used as an internal standard for accurate quantification.

## Results and Discussion

We developed a rapid, sensitive method for detecting and differentiating BoNT serotypes A, B, E, and F. Each BoNT serotype recognizes and cleaves a unique site on either SNAP-25 or VAMP-2 (Figure 1). We synthesized the specific portions of SNAP-25 and VAMP that are substrates for the 4 BoNT serotypes that commonly cause human botulism (serotypes A, B, E, and F). We used the endopeptidase activity to detect and differentiate the specific BoNT serotype by allowing the BoNT to cleave its specific peptide substrate and detecting the cleavage products by mass spectrometry.

The substrate peptides were designed to be the same as the sequences of those portions of the natural SNAP-25 (for BoNT-A and -E) or VAMP (for BoNT-B and -F) that are recognized and cleaved, except some modifications were made for BoNT-A and -B. For BoNT-A, the peptide from SNAP-25 that includes serine-187 to glycine-206 is required for cleavage at glutamine-196. Schmidt et al. found that replacing lysines 189 and 201 with arginines showed enhanced cleavage by BoNT-A (*14*). We also found an increase in the amount of BoNT-dependent cleavage products detected in the modified peptides. The portion of VAMP-2 required for cleavage by BoNT-B at glutamine-75 is leucine-59 to lysine-93, and the portion required for cleavage by BoNT-F at glutamine-57 is from alanine-36 to serine-74. The portion of SNAP-25 from isoleucine-156 to aspartic acid-186 is required for cleavage between arginine-180 and isoleucine-181 by BoNT-E. We biotinylated the N and C termini of the substrate peptide for BoNT-A so that the product peptides of interest can be easily purified from complex matrices. After final peptide sequences are determined, we plan to biotinylate all substrate peptides.

**Figure A1:**
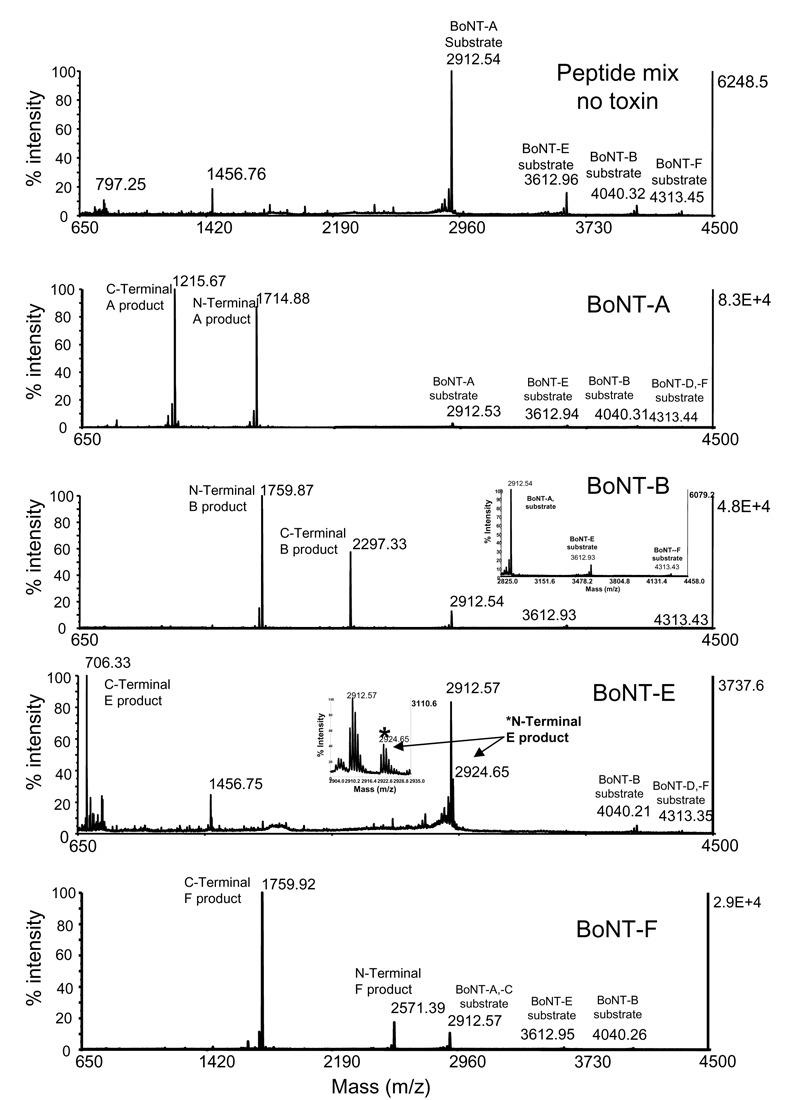
Matrix-assisted laser desorption time-of-flight mass spectrometry (MALDI-TOF-MS) spectrum of botulinum neurotoxin (BoNT) reactions. The top panel shows the MALDI-TOF-MS spectrum of the mixture of substrate peptides with no toxin present. The second panel shows this same mixture of substrate peptides and BoNT-A. The third, fourth and fifth panels show the mixture of substrate peptides with BoNT-B, -E, and –F, respectively. The insert mass spectrum for BoNT-E distinguishes the BoNT-dependent N-terminal product from the BoNT-A substrate peptide.

The method was multiplexed by combining all 4 substrate peptides for the BoNT serotypes A, B, E, and F into a sample that contained various levels of a single BoNT serotype or no toxin. The expected product peptide masses and the masses of the substrate peptides are shown in Figure 2. The product peptides for each specific BoNT serotype can be easily distinguished by their mass. Figure A1 shows typical results for each of the reaction mixtures containing the 4 substrate peptides incubated with only the reaction buffer (a blank) or with 1 of the BoNT serotypes. Each of the BoNT serotypes yielded only the expected cleavage products from its respective substrate peptides, indicating that this method can easily detect and differentiate active BoNT serotypes. No cleavage was observed in the reactions that did not contain BoNT. Additionally, even at this relatively high toxin level, no cross-reactivity was seen between the toxin types; only the expected peptide cleavage reactions were observed.

We also tested sensitivity of the method for each single toxin serotype with a single substrate peptide. Since enzymatic reactions tend to be concentration dependent, this testing was accomplished in 2 ways. First, we determined the sensitivity on the basis of the lowest absolute amount of toxin that could be detected by the Endopep-MS method in a 20-μL reaction; second, we determined the lowest concentration per milliliter that could be detected by this method. The first approach indicates the minimum amount of toxin that needs to be present, and the second indicates the minimum concentration of toxin in a sample. For BoNT serotypes A, B, and F as little as 0.01 MLD_50_ yielded sufficient quantities of product peptides to be clearly detected by MALDI-TOF-MS. This figure is 100× lower in the absolute amount of toxin than that required by the mouse bioassay. For BoNT-E, product peptides could be detected by MALDI-TOF-MS with as little as 0.08 MLD_50_. The analytic sensitivity of the method was then tested to determine the lowest measurable concentration of the toxin. An aliquot of a 1-mL sample that contained 100 MLD_50_ to 0.31 mouse LD_50_ in water was tested by both the MALDI-TOF-MS and HPLC-ESI/MS/MS methods. Reactions were also allowed to proceed for 4 h and for 16 h. The 4-h reactions for BoNT-A, -B, and -E showed the sensitivity by MALDI-TOF-MS detection of the product peptides of 1.2 mouse LD_50_/mL for BoNT-A and -B and 6.2 mouse LD_50_/mL for BoNT-E. After 16-h reactions, the sensitivity was 0.62 mouse LD_50_/mL for BoNT-A and -B, 0.31 mouse LD_50_/mL for BoNT-E, and 6.2 mouse LD_50_/mL for BoNT-F. Using HPLC-ESI/MS/MS to analyze these same low toxin samples, we found that after 16-h incubation, the detection limits were 0.62 mouse LD_50_/mL for BoNT-A and -B, <0.31 MLD_50_/mL for BoNT-E, and 0.62 MLD_50_/mL for BoNT-F. These data indicate that the Endopep-MS method is very sensitive with respect to the amount and concentration of toxin and that methods that can concentrate active toxins into smaller reaction volumes (thus yielding higher concentration) will result in lower limits of detection.

**Figure 3:**
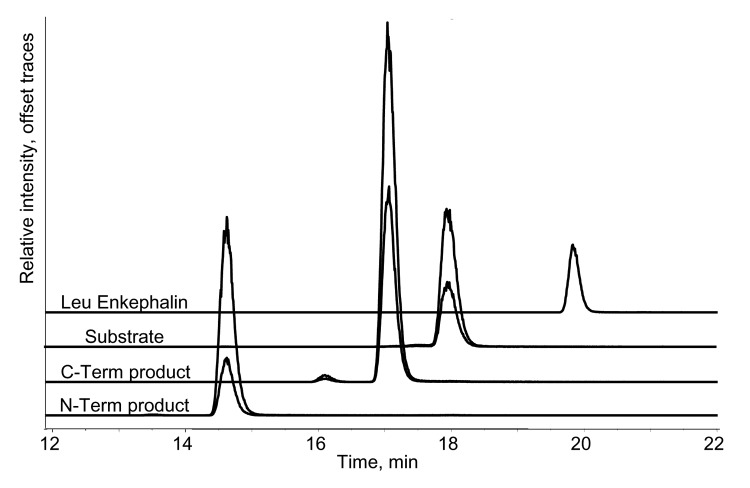
High-performance liquid chromatography–electrospray ionization-tandem mass spectrometry chromatogram showing the botulinum neurotoxin (BoNT)-A substrate and product ions (CT, C-terminal; NT, N-terminal) from a reaction with 25 mouse lethal dose (MLD)50 BoNT-A. Each peptide has both a quantification ion (top trace) and a verification ion (lower trace). Isotopically labeled standards are added (traces not shown) as internal standards for quantification. The labeled peptides co-elute with their nonlabeled counterparts and are distinguishable by mass. Leucine enkephalin was included as a secondary reference compound and only 1 ion was monitored.

The HPLC-ESI/MS/MS technique to quantitatively detect and differentiate BoNT activities is highly selective. Correct identification of the BoNT product peptides depends on both a retention time match, with respect to standards, and on a chemical-specific fragmentation (a precursor to product ion multiple reaction monitoring [MRM] transition) monitored by tandem MS. To further enhance selectivity, 2 separate MRM transitions can be monitored for each peptide. In addition, our HPLC-ESI/MS/MS technique is very sensitive. Quantification of the BoNT product peptides is achieved by using stable isotope-labeled internal standards that have the same sequence as the native product peptides but are labeled with ^13^C. Figure 3 shows typical HPLC-ESI/MS/MS chromatograms obtained during the quantification of the activity of BoNT-A, and a standard curve for both of the product peptides. The amount of product peptide was then correlated with the amount of toxin that yielded the product peptides. Standard curves between 10 and 1,000 MLD_50_ were prepared, and individual spiked samples were run as unknown samples to determine the accuracy and precision of the method. The spiked samples were prepared at 16, 32, 65, and 125 MLD_50_; 2 samples were run in duplicate at each spike level giving a total of 4 measurements. The accuracy and precision of these measurements, shown in the Table, were good; relative standard deviations were <2%–5%. This method is the first that can accurately quantify BoNT enzymatic activity. Identical HPLC-ESI/MS/MS strategies can be used to quantify each of the BoNT serotypes.

**Table:** Accuracy of quantification of BoNT-A*

Spike level (mouse LD_50_)	Measured concentration mean SD (N = 4)
16	19.85 ± 1.71
32	33.75 ± 3.24
65	68.05 ± 5.07
125	120.78 ± 2.66

*BoNT, botulinum neurotoxin; LD, lethal dose; SD, standard deviation.

The Endopep-MS method has many possible applications. Beyond using the Endopep-MS method for identifying the BoNT serotype in a clinical, food, or environmental sample, standardizing BoNT activity in samples used for clinical treatment or research activities may be possible. The standardization of BoNT, both the amount of 150-kDa toxin and the activity of a standard solution, is of great importance in the medical use of BoNT (*16*). A possible strategy for standardizing BoNT includes correlating the activity obtained by the mouse bioassay to the Endopep-MS method. Additionally, it may be possible to correlate this activity to an absolute amount of toxin that is determined in a similar fashion as was done for apolipoprotein A-1 (*17*).

Endopep-MS currently has limitations. Mass spectrometry equipment is expensive and requires a high level of technical expertise for optimal operation. The method still needs to be tested in a wide variety of clinical and food samples and may require the use of protease inhibitors and affinity chromatography to partially purify and concentrate the toxin. Also, the Endopep-MS method needs to be validated against the mouse bioassay. The method also has several strengths. It is rapid, and in simple matrices, such as water and buffer, it can obtain similar sensitivities to the mouse in <5 h. Samples can be prepared for the reactions in minutes; incubation times of only 4 h yield sensitivity close to the mouse bioassay, and MALDI-TOF mass spectra can be collected in <1 min. Samples can also be batched so that 50 samples can be analyzed in <6 h. The HPLC-ESI/MS/MS analysis is somewhat slower than the MALDI-TOF-MS, and each sample requires 30 min. However, this process has been automated, and batches of samples can run unattended so that 40 samples a day can be processed and run by HPLC-ESI/MS/MS. The method is well suited for multiplexing and can not only detect but also differentiate toxin type in a single analytic run.

## Conclusions

We developed a method based on the unusually specific endopeptidase activity of BoNT that uses highly selective mass spectrometry analysis to detect and differentiate BoNT serotypes A, B, E and F. This method is rapid and sensitive. When the endopeptidase reactions are allowed to proceed for 16 h, it is highly sensitive; 100× more sensitive in absolute amounts of active toxin for BoNT-A, -B, and -F and >10× more sensitive than the mouse bioassay for BoNT-E. Since each BoNT serotype has a unique cleavage site on a substrate peptide, this analysis lends itself well to multiplexing. We multiplexed the analysis and showed that no cross-reactivity occurs within the 4 BoNT serotypes. The Endopep-MS method should be very useful to rapidly detect and differentiate active BoNT in a variety of clinical, food, or environmental samples to quickly establish the serotype(s) and aid in identifying a source during an outbreak. It may also prove useful for standardizing BoNT enzymatic activity for preparations used as clinical treatments or for research activities. This type of approach may also prove useful for detecting other proteolytic toxins.
